# Scalable and number-controlled synthesis of carbon nanotubes by nanostencil lithography

**DOI:** 10.1186/1556-276X-8-281

**Published:** 2013-06-11

**Authors:** Jungwook Choi, Kisik Koh, Jongbaeg Kim

**Affiliations:** 1 School of Mechanical Engineering, Yonsei University, 50 Yonsei-ro, Seodaemun-gu, Seoul 120-749, Republic of Korea

**Keywords:** Carbon nanotube, Stencil lithography, Scalable growth, Number-controlled growth

## Abstract

Controlled synthesis and integration of carbon nanotubes (CNTs) remain important areas of study to develop practical carbon-based nanodevices. A method of controlling the number of CNTs synthesized depending on the size of the catalyst was characterized using nanostencil lithography, and the critical dimension for the nanoaperture produced on a stencil mask used for growing individual CNTs was studied. The stencil mask was fabricated as a nanoaperture array down to 40 nm in diameter on a low-stress silicon nitride membrane. An iron catalyst used to synthesize CNTs was deposited through submicron patterns in the stencil mask onto a silicon substrate, and the profile of the patterned iron catalyst was analyzed using atomic force microscopy. The feasibility toward a scalable, number-, and location-controlled synthesis of CNTs was experimentally demonstrated based on the diameter and geometry of the apertures in the stencil mask.

## Background

Intensive research has been performed on carbon nanotube (CNT)-integrated microdevices and nanodevices to take advantage of the remarkable thermal, mechanical, electrical, and electromechanical properties of CNTs [1]. Examples of such devices include nanoelectronic devices and optoelectronic components [2-4], actuators and oscillators [5-7], memory devices and switches [8,9], and mechanical, chemical, biological, and thermal sensors [10-13]. Controlling the number of CNTs synthesized and their specific placement on nanostructures and microstructures is critical to using the inherent properties of massively parallel-integrated CNTs for practical device applications. However, previously reported methods of integrating CNTs in CNT-based devices are low-throughput methods such as dispersion of CNTs followed by electron beam lithography patterning [10], dielectrophoresis [14-17], and pick-and-place manipulation [18]. Although the assembly of individual CNTs at specific locations has previously been demonstrated using such methods, high-throughput batch fabrication has not been feasible over a large area because of time-consuming, labor-intensive processes. Chemical vapor deposition (CVD) is scalable over a large area, so it is an attractive alternative for directly integrating individual CNTs into practical device applications. Accordingly, various methods of patterning nanocatalysts have been developed using electron beam lithography [19], nanoimprinting [20], polystyrene nanospheres [21], anodic aluminum oxide nanotemplates [22], nanocontact printing [23], and topographical contact holes [24] to synthesize individual CNTs under controlled conditions.

We used nanostencil lithography as a method of patterning a nanocatalyst to demonstrate and characterize number- and location-controlled synthesis of CNTs. Nanostencil lithography has been widely used to fabricate various nanopatterns [25-28], nanoparticles [29,30], and nanowires [31], and it is advantageous because it consists of a series of simple fabrication steps and because the stencil mask is reusable. Moreover, the degree of contamination of the catalyst during patterning might be negligible in nanostencil lithography because patterning is conducted under vacuum without need for a photoresist, a solvent, or chemicals used for patterning and etching [32], thereby producing a residue-free catalyst suitable for CVD synthesis of CNTs. We used focused ion beam (FIB) milling on a silicon nitride membrane to fabricate nanostencil aperture arrays down to 40 nm in diameter, and the stencil mask was used to pattern a submicron iron catalyst. The thickness and width of the iron catalyst deposited through the stencil mask were analyzed using atomic force microscopy (AFM). The number of synthesized CNTs could be controlled based on the size of the aperture in the stencil mask, and individual CNTs were synthesized over a large area.

## Methods

**Figure 1:**
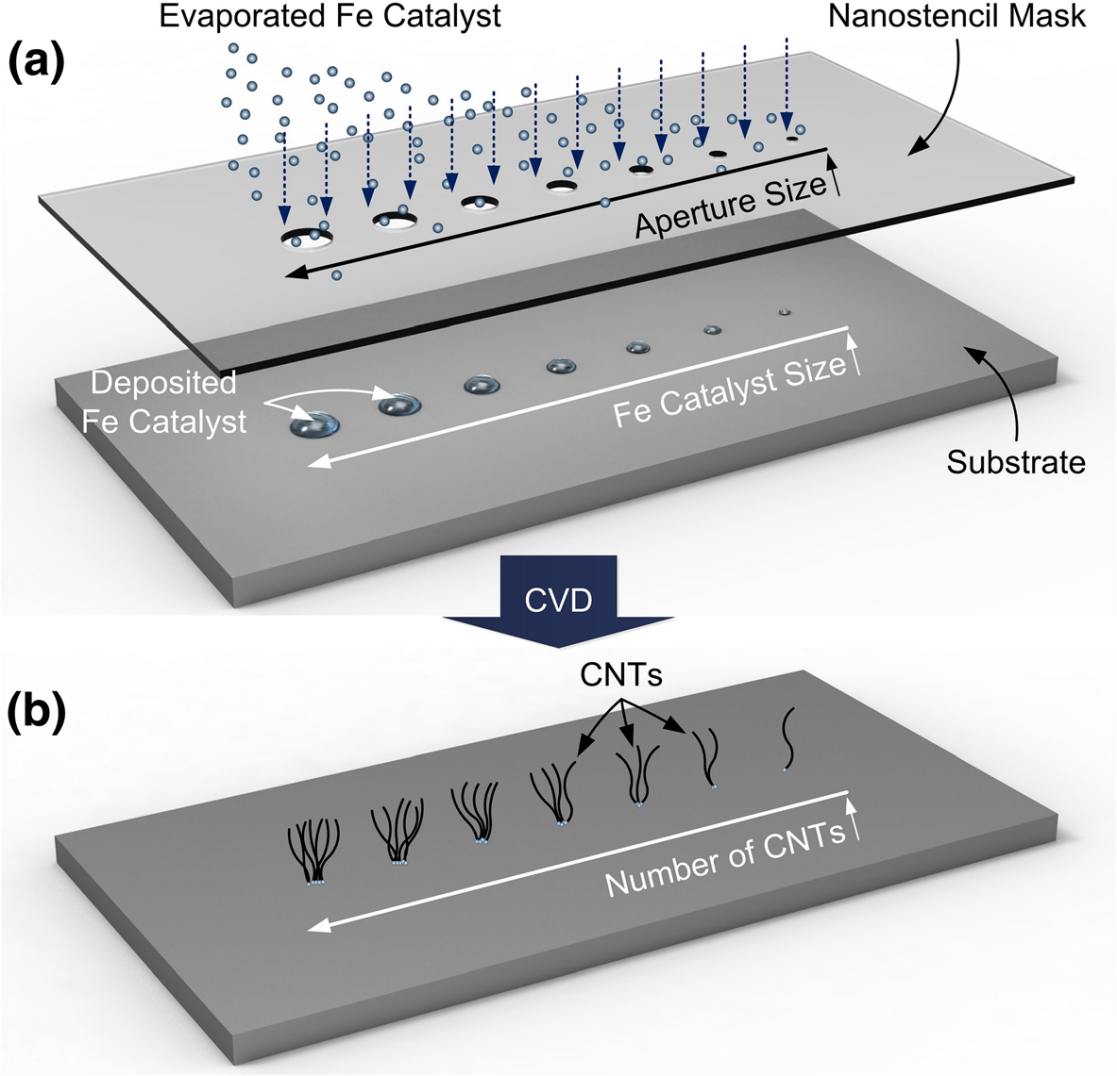
**The experimental procedure of nanostencil lithography and subsequent CVD to synthesize number- and location-controlled CNTs.** (**a**) Evaporated iron catalyst is deposited through nanoapertures onto the substrate. The size of the deposited iron catalyst decreases with decreasing aperture size. (**b**) CNTs are synthesized on patterned catalyst, and the number of CNTs synthesized is controlled based on the size of catalyst pattern. Thus, number-controlled, location-specific synthesis of parallel-integrated CNTs can be achieved over a large area.

An illustration of the nanostencil lithography used to pattern the nanocatalyst and the subsequent CNT synthesis are shown in Figure 1. The stencil mask was aligned on the substrate, and the iron catalyst was deposited through stencil apertures onto the substrate (Figure 1a). Thus, the overall process used to pattern a submicron catalyst is much simpler than conventional resist-based methods such as lift-off or top-down etching [31]. Any desired patterns of individual CNTs could be produced based on the geometrical design of the stencil apertures. Moreover, it is expected that decreasing the size of the apertures in the stencil mask would decrease the size of the catalyst deposited onto the substrate, which would in turn decrease the number of synthesized CNTs, as shown in Figure 1b. Electron beam evaporation was performed under 5 × 10^−5^ Torr to deposit an iron catalyst whose nominal thickness was 5 nm. The substrate was then loaded into a tube furnace for CVD in order to synthesize individual CNTs. A rotary vane pump was used to pump down the furnace to a base pressure, and the furnace was then purged with 100 sccm of nitrogen. When the temperature inside the furnace reached 700°C, 100 sccm of ammonia was introduced for 40 min to pretreat the iron catalyst. Synthesis of the CNTs was then initiated by flowing 30 sccm of acetylene into the furnace for 10 min, and the furnace was cooled to room temperature under 100 sccm of flowing nitrogen. We used identical CVD conditions in every experiment presented here to verify size dependency of the catalyst on the number of CNTs since different CVD temperatures, composition of gases, and flow rate would also affect the number of CNTs grown [33,34].

**Figure 2:**
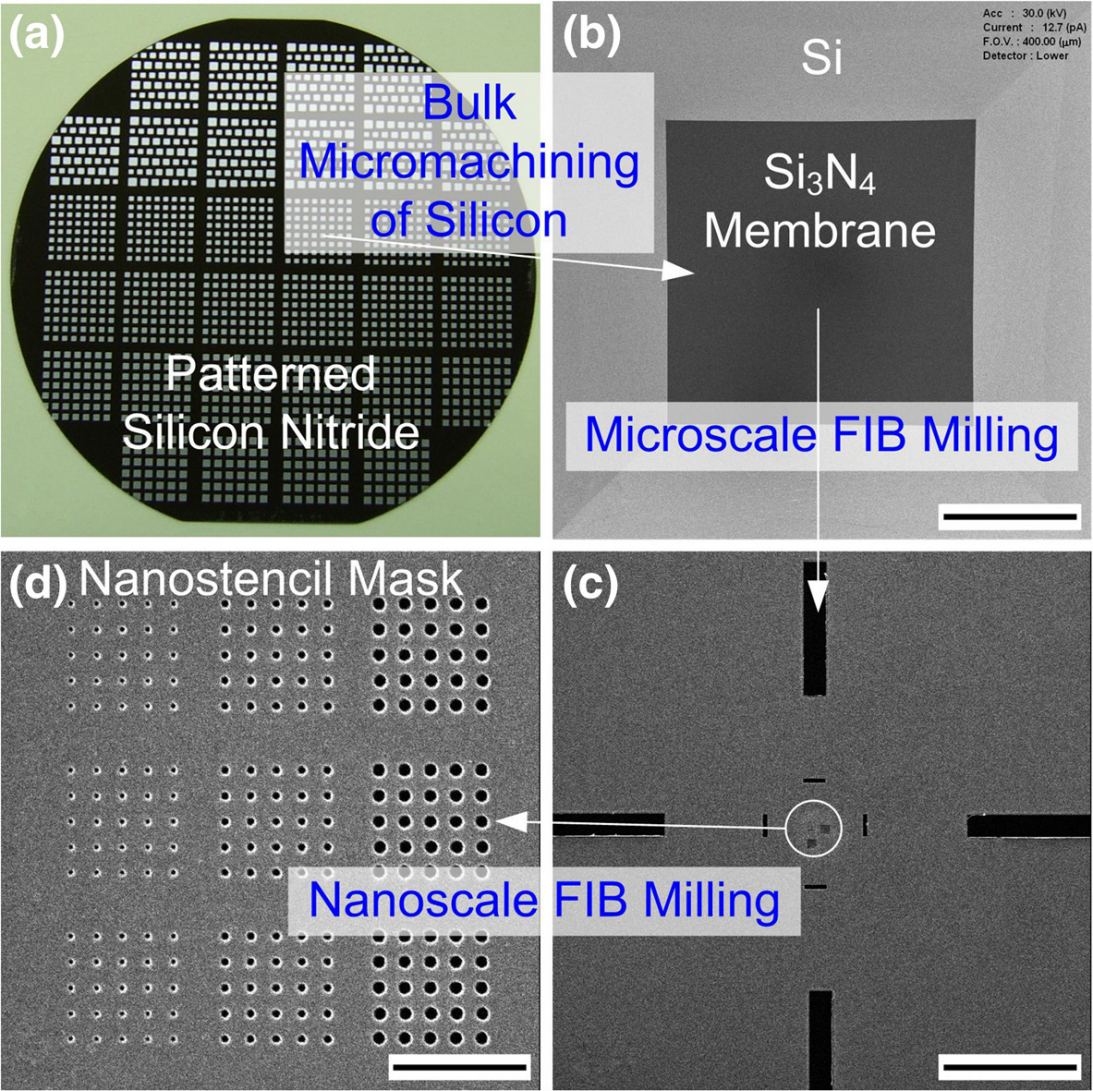
**Sequential images of fabrication of nanostencil mask.** (**a**) Low-stress silicon nitride film (50-nm thick) was deposited and patterned onto both sides of a 4-in. silicon wafer. (**b**) Silicon nitride membranes were released using KOH to anisotropically etch silicon. (**c**, **d**) Microscale and nanoscale FIB milling were performed on the membranes to form reference marks and apertures. Scale bars shown in (**b**), (**c**), and (**d**) are 100, 30, and 3 μm, respectively.

Bulk micromachining and FIB milling were used to fabricate the stencil masks on a 4-in. silicon wafer. A 50-nm-thick low-stress silicon nitride film was deposited onto both sides of the wafer, and photolithography and reactive-ion etching were subsequently performed to define the membranes. The membranes were then released using KOH to anisotropically etch the silicon at 80°C (see Additional file 1: Figure S1 for the detailed fabrication process). The patterned silicon nitride on the 4-in. silicon wafer is shown in Figure 2a, and the scanning ion microscopy (SIM) image of the fabricated membrane is shown in Figure 2b. FIB milling was used to fabricate nanoapertures on the membranes. FIB has been widely used as versatile method of modifying semiconductor circuits, etching nanoholes, and fabricating nanostructures [35-37]. Before the patterns were defined on the membranes, sputtering was performed to deposit a 5-nm-thick layer of Pt-Pd alloy onto the membranes in order to prevent charging during FIB milling. As shown in Figure 2c, microsquares were first patterned as reference marks for future alignment with prefabricated microstructures on the substrate. The nanoapertures were then cut off using FIB milling at 30 keV of ion acceleration energy and at 1 pA of ion beam current [38], and the diameter of the apertures was defined by controlling the ion dose, as shown in Figure 2d. FIB milling was used to form the diverse range of geometrical shapes and sizes of the apertures (see Additional file 1: Figure S2 for examples of various nanoapertures), and the patterns could be transferred to target electrodes or substrates in order to control the integration of CNTs. In addition, the fabricated stencil masks could be reused many times without sustaining any damage [31].

## Results and discussion

**Figure 3:**
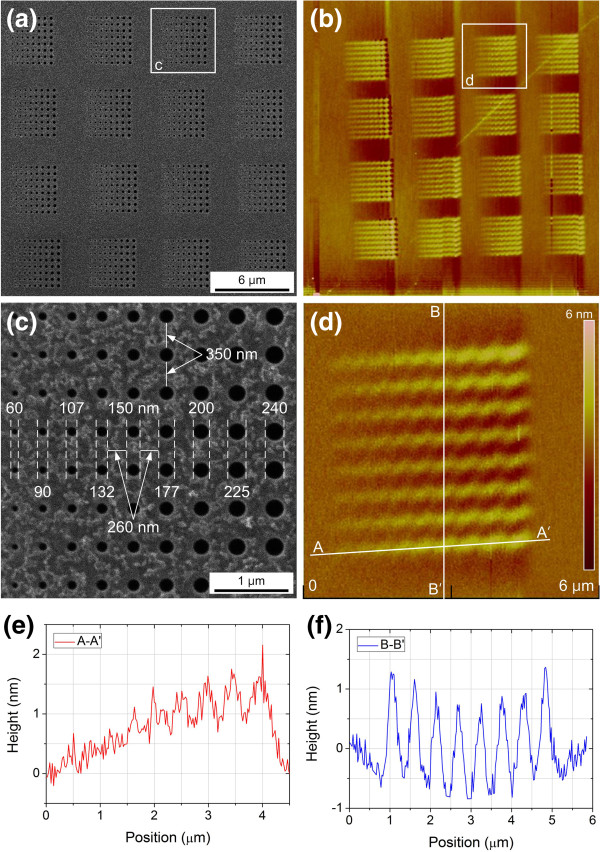
**Correlation between aperture diameter and deposited iron catalyst.** (**a**) SIM image of the stencil mask fabricated with 1,152 nanoapertures. (**b**) Tapping-mode AFM image of the iron catalyst deposited onto the substrate through the stencil mask. (**c**, **d**) Enlarged SIM and AFM images of the apertures and patterned iron catalyst shown in (**a**) and (**b**), respectively. Diameter of the apertures was 60 to 240 nm, and horizontal spacing between apertures was 260 nm. (**e**, **f**) Cross-sectional height profiles for iron catalyst deposited along lines indicated by A-A' and B-B' in (**d**). Height of the deposited catalyst increases with increasing diameter of aperture, and thickness of the iron catalyst deposited through 177-nm aperture is 1.6 to 1.7 nm.

The widths and heights of the iron catalysts deposited through the nanostencil apertures of various diameters were analyzed using AFM. A total of 1,152 aperture arrays (4 × 4 arrays each consisting of 8 × 9 apertures) were fabricated in a stencil mask, as shown in Figure 3a, and the iron catalysts were deposited through the aperture arrays of the stencil onto the silicon substrate. All of the aperture patterns were transferred to the iron catalyst, as shown in the AFM image in Figure 3b. The enlarged image of the apertures and the corresponding patterned iron catalysts are shown in Figure 3c,d, respectively. The diameter of the apertures varied from 60 to 240 nm, and the horizontal spacing between the adjacent apertures was 260 nm, as shown in Figure 3c. Figure 3d exhibits an AFM image of an 8 × 9 array iron catalyst deposited through the stencil mask, and the corresponding height profiles measured along the A-A' and B-B' lines shown in Figure 3d are plotted in Figure 3e,f, respectively. The cross-sectional height measured along the A-A' line shown in Figure 3d gradually increases, as shown in Figure 3e, which implies that the amount of iron catalyst deposited through the nanostencil apertures increases with increasing aperture diameter. The effect of aperture size on the transferred pattern has previously been demonstrated for metallic nanowire fabrication [31]. In addition, the boundary between neighboring iron catalysts is obscure because of blurring, which could be decreased by decreasing the size of the gap between the stencil and the substrate, decreasing the deposition rate, decreasing the temperature of the substrate during evaporation [39], or by a combination thereof. The boundary of the height profile measured along the B-B' line shown in Figure 3f is clearer than that of the height profile measured along the A-A' line despite blurring since the vertical spacing (350 nm) between each aperture used to deposit the iron catalyst along the B-B' line is larger than the horizontal spacing (260 nm) along the A-A' line. The thickness and the average diameter of the iron catalyst patterns deposited through the 177-nm-diameter apertures were 1.6 to 1.7 nm and 449 nm, respectively, which revealed that significant blurring existed during the pattern transfer.

**Figure 4:**
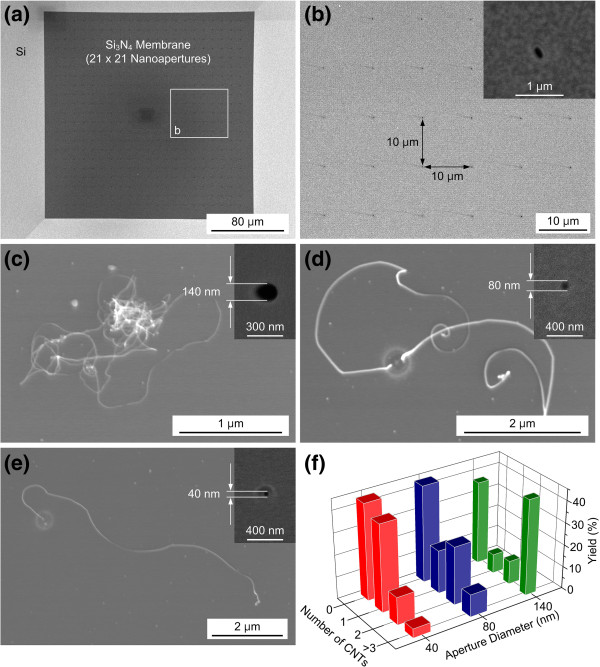
**Number-controlled growth of CNTs by using apertures with different diameters.** (**a**, **b**) SIM images of 21 × 21 nanoapertures in a stencil mask consisting of 140-nm-diameter apertures and 10-μm spacing between each aperture. The inset in (**b**) shows an enlarged view of the aperture shown in (**a**). (**c**, **d**, **e**) SEM images of CNTs synthesized using apertures of various diameters. Some CNTs (**c**), mainly double CNTs (**d**), and individual CNT (**e**) were synthesized through apertures whose diameters were 140, 80, and 40 nm, respectively. The insets show the apertures used to synthesize CNTs. (**f**) The number of CNTs synthesized was strongly dependent on the diameter of the nanostencil aperture. Yield was 39.6% for individual CNTs synthesized using 40-nm-diameter aperture.

The number of CNTs synthesized using CVD and apertures of various diameters was analyzed. Some 21 × 21 apertures whose diameters were 140, 80, or 40 nm were fabricated (Figure 4a) for the experiments, and the spacing between each aperture was 10 μm to prevent any possibility of catalyst pattern interference due to blurring between neighboring apertures, as shown in Figure 4b. The ion doses used during FIB milling to produce the 140-, 80-, and 40-nm apertures were 1.99 × 10^18^, 9.95 × 10^17^, and 3.98 × 10^17^ ions cm^−2^, respectively. As shown in the scanning electron microscopy (SEM) images in Figure 4c,d,e, the number of CNTs synthesized at a specific location can be controlled by designing the diameter of the nanostencil aperture. A few CNTs were synthesized on the iron catalyst that had been deposited through the 140-nm-diameter aperture (Figure 4c), and the number of CNTs decreased to two for the iron catalyst that had been deposited through the 80-nm-diameter aperture (Figure 4d). Directly synthesizing individual CNTs onto a desired site is highly preferred in order to use the unique material properties of individual CNTs for various applications and prevent interactions between CNTs. An individual CNT was synthesized when the 40-nm-diameter aperture was used to pattern the iron catalyst, as shown in the SEM image in Figure 4e. The correlation between the aperture diameter and the number of CNTs synthesized under the experimental conditions is summarized in Figure 4f. The number of CNTs obviously decreased with decreasing aperture diameter. For example, although 39.6% of the CNTs synthesized through the 40-nm-diameter aperture were individual CNTs, the yield for the growth of single CNTs decreased to 19.6% and 8.7% when the 80- and 140-nm-diameter apertures were used, respectively. Furthermore, the yield for the synthesis of two CNTs using the 80-nm-diameter aperture was more than twice compared to that for the synthesis of two CNTs using the other two apertures. Hence, there is a high chance of controlling the number of CNTs synthesized by adjusting the diameter of the aperture used in the nanostencil mask. More results for the number of CNTs synthesized using various aperture diameters are shown in Additional file 1: Figure S3. The diameter of the synthesized CNTs was 10 to 30 nm, which indicates that they exhibited a multiwalled structure. It also reveals that the iron catalyst was agglomerated into a size similar to the diameter of CNTs in CVD temperature of 700°C [40-42]. No CNTs were found on approximately 40% of the catalytic sites produced using the three different aperture sizes. It could possibly be from the size deviation in each catalyst pattern, and this would be improved by enhancing the mechanical stability of the stencil mask through the design of corrugated structures [43], by increasing the directionality and the nominal thickness of the iron catalyst, or by introducing a buffer layer such as aluminum oxide between the catalyst and the silicon substrate to prevent the possible formation of iron silicide. Although our method is not perfect, it retains higher throughput, yield, and scalability than other serial processes used to integrate individual CNTs on specific sites, such as electron beam lithography on dispersed CNTs [10], pick-and-place manipulation [18], and localized synthesis on microheaters [44]. The integrity and throughput of our method are also superior to those of dielectrophoretic assembly [14-17], which is frequently used to integrate individual CNTs. CNTs should be immersed and sonicated in an aqueous solution for dielectrophoresis. This process usually contaminates the CNTs, deteriorating their unique material properties. In addition, high-throughput integration of individual CNTs is limited since each of the electrodes where the CNTs are assembled should be electrically accessed to induce dielectrophoresis. To fabricate CNT-based two-terminal devices using our approach, horizontal alignment of CNTs might be necessary, and the orientation of CNTs could be aligned to the applied electric field [45], magnetic field [46], and direction of gas flow [47]. Nanostencil lithography could be extended to control the number of various one-dimensional nanomaterials that are grown and the specific sites where they are grown by depositing other catalysts such as gold for silicon, gallium nitride, or zinc oxide nanowires. Since these one-dimensional nanomaterials have unique characteristic, for example, ZnO nanowire array exhibits giant optical anisotropy due to high aspect ratio, subwavelength diameter, and high permittivity [48], the proposed position- and number-controlled synthesis approach could be useful to realize nanomaterial-based devices with enhanced functionalities and mass producibility.

## Conclusions

We fabricated stencil masks containing nanoaperture arrays down to 40 nm in diameter in order to characterize the relation between the size of the patterned catalyst and the number of CNTs that were subsequently synthesized on the catalyst. The nanostencil mask was fabricated by first forming a low-stress silicon nitride membrane on a silicon substrate. FIB milling was subsequently used to generate nanoapertures on the membrane. The iron catalyst used to synthesize the CNTs was then deposited through the nanoapertures onto the silicon substrate. The diameter of iron catalyst was larger than that of the aperture because of blurring, and the thickness of the catalyst decreased with decreasing aperture diameter. Accordingly, the number of CNTs could be controlled by controlling the diameter of the aperture, and the iron catalyst patterned with the 40-nm-diameter aperture on the stencil mask was used to synthesize an individual CNT at the desired sites. The demonstrated scalable, number- and location-controlled synthesis of CNTs is potentially applicable to massively parallel integration of single CNTs on each of the desired locations and may enhance the producibility and yield of CNT-based functional devices.

## Abbreviations

AFM: Atomic force microscopy; CNT: Carbon nanotube; CVD: Chemical vapor deposition; FIB: Focused ion beam; SEM: Scanning electron microscopy; SIM: Scanning ion microscopy.

## Competing interests

The authors declare that they have no competing interests.

## Authors' contributions

JC analyzed the experimental data and drafted the manuscript. KK carried out the experiments. JK initiated and supervised the work. All authors read and approved the final manuscript.

## Supplementary Material

Additional file 1 **Supporting information on the scalable and number-controlled synthesis of carbon nanotubes by nanostencil lithography.** Includes a detailed fabrication process of the nanostencil mask, images of the various nanostencil apertures, and images of the synthesized CNTs. Click here for file
