# Supplementary material for: Scalable and number-controlled synthesis of carbon nanotubes by nanostencil lithography
Source: Nanoscale Res Lett. 2013 Jun 11;8(1):281. doi: 10.1186/1556-276X-8-281 (PMC3683346; doi:10.1186/1556-276X-8-281)
Supplement: Additional file 1 — Supporting information on the scalable and number-controlled synthesis of carbon nanotubes by nanostencil lithography. Includes a detailed fabrication process of the nanostencil mask, images of the various nanostencil apertures, and images of the synthesized CNTs. [file 1556-276X-8-281-S1.pdf]

Supporting Information

Scalable and number-controlled synthesis of carbon nanotubes by nanostencil lithography

Jungwook Choi, Kisik Koh, and Jongbaeg Kim*

School of Mechanical Engineering, Yonsei University

50 Yonsei-ro, Seodaemun-gu, Seoul 120-749, Republic of Korea

*E-mail address: kimjb@yonsei.ac.kr

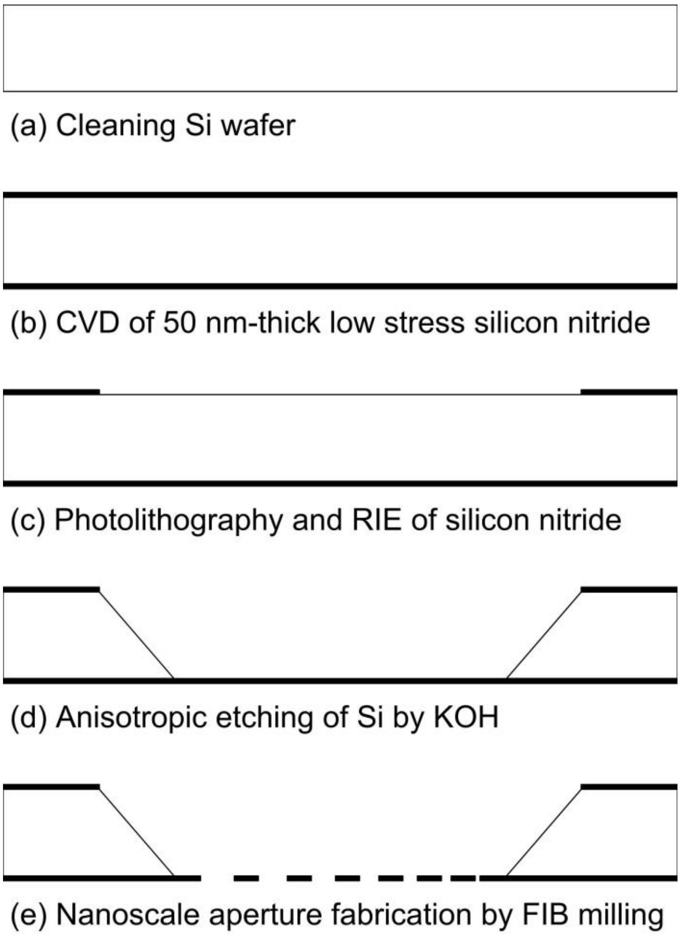

Figure S1. Detailed fabrication process of the nanostencil mask.

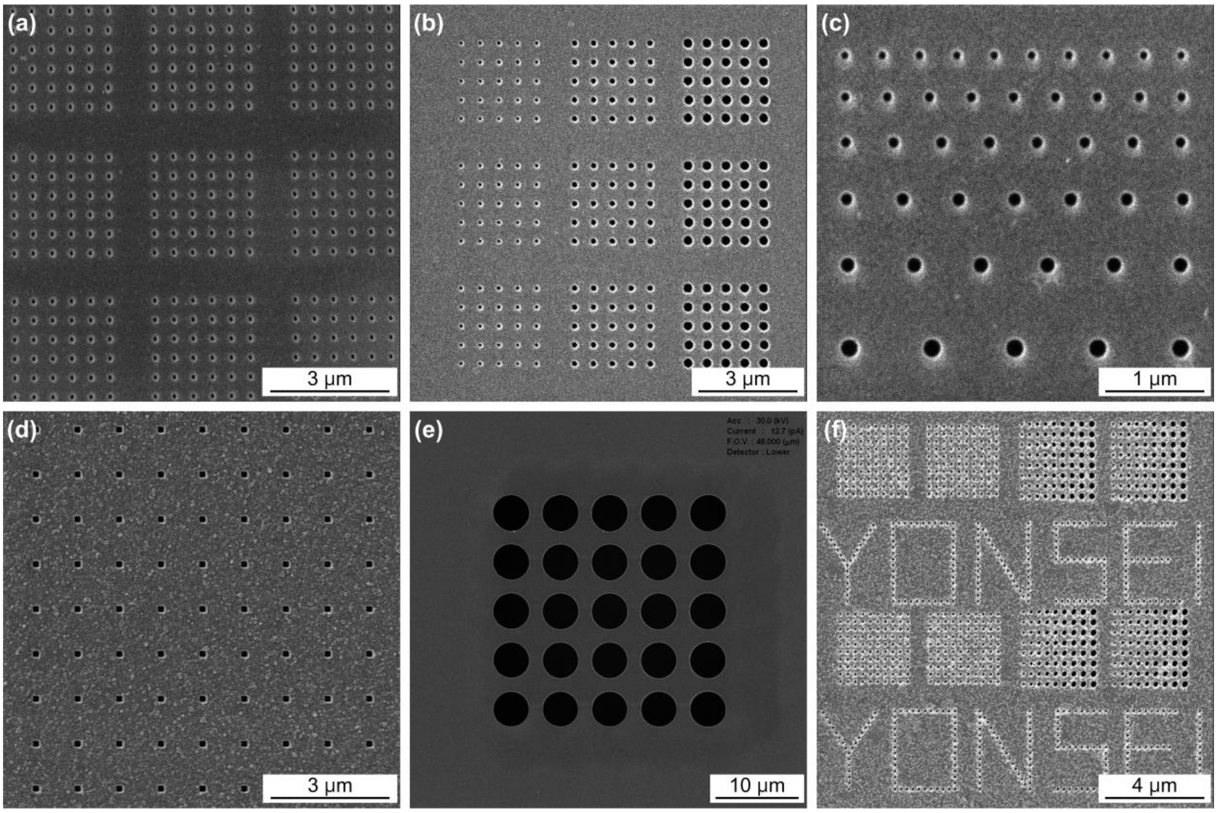

Figure S2. Examples of stencil masks with various nanoscale apertures. (a) Nanohole arrays with same diameter. (b) Nanohole arrays with increasing diameter. (c) Nanohole array with increasing diameter and spacing. (d) Rectangular-shape nanohole array. (e) Microhole array with same diameter. (f) Arbitrary patterns of nanoholes.

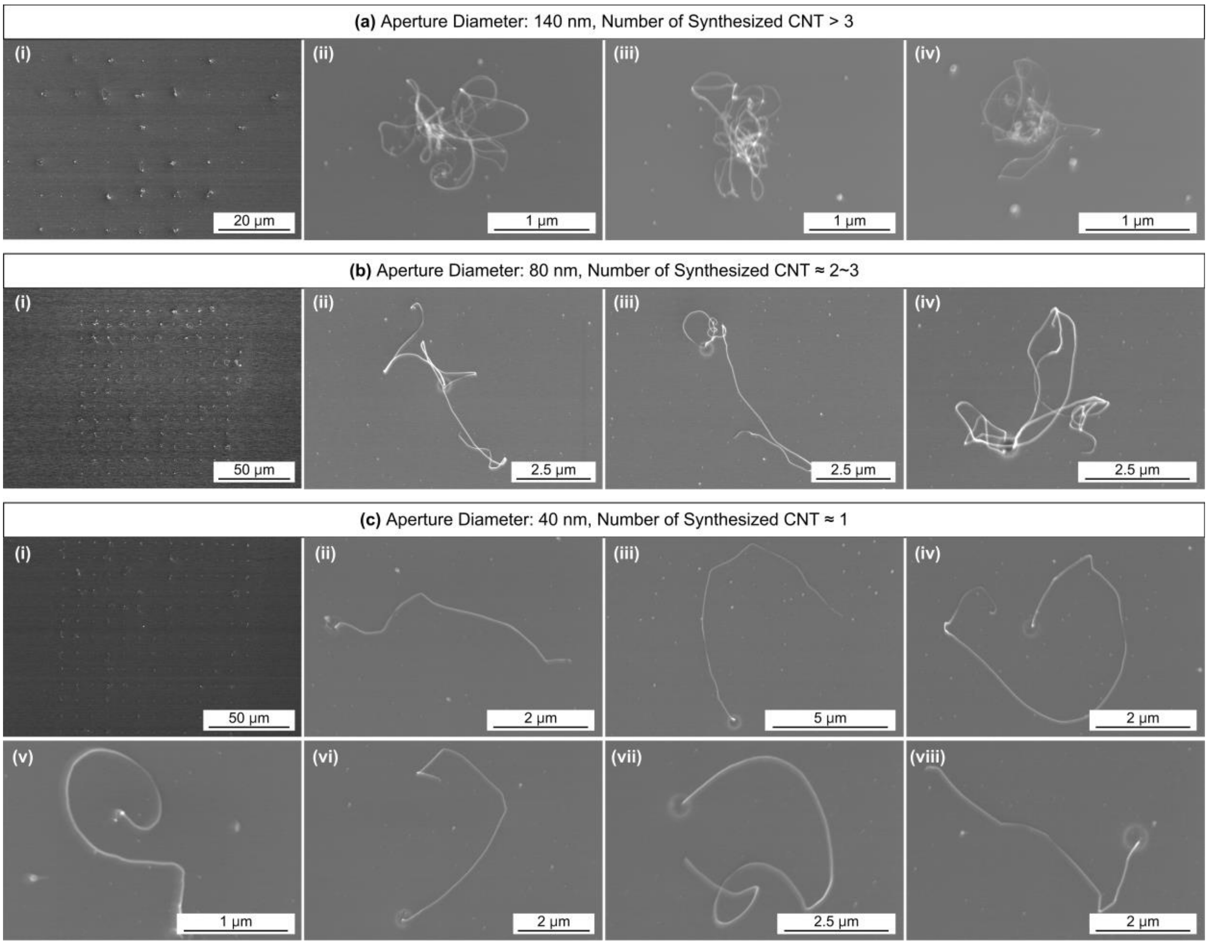

Figure S3. Exemplary SEM images of synthesized CNT according to the aperture diameter.
